# miR34a: a master regulator in the pathogenesis of bronchopulmonary dysplasia

**DOI:** 10.15698/cst2018.02.1224

**Published:** 2018-01-09

**Authors:** Pragnya Das, Mansoor Ali Syed, Dilip Shah, Vineet Bhandari

**Affiliations:** 1 Section of Neonatology, Department of Pediatrics, Drexel University College of Medicine, Philadelphia 19102.; 2 Department of Biotechnology, Jamia Millia Islamia, New Delhi, India 110025.; 3 St. Christopher’s Hospital for Children, Philadelphia, PA 19134.

**Keywords:** miR34a, BPD, hyperoxia, lungs, angiopoietin1, p53, SIRT1, surfactant

## Abstract

Bronchopulmonary dysplasia (BPD) is the most common chronic lung disease in infants with lifelong pulmonary and neurodevelopmental consequences. The pathogenesis of BPD is contributed by genetic and environmental factors; among the latter, a critical contributor is exposure of the developing lung to hyperoxia. We have recently reported (Nat Comm 8:1173) that hyperoxia exposure in our *in vitro* and *in vivo *modeling systems of hyperoxia-induced lung injury (HALI) and BPD leads to an upregulation of the microRNA (miR) 34a. Utilizing genetic loss- and gain- of function strategies, we show that miR34a inhibition ameliorates the pulmonary phenotype of BPD (including BPD-associated pulmonary hypertension), at least in part, via one of the downstream targets of miR34a, namely Angiopoietin1/Tie 2 signaling. In addition, we demonstrate translational clinical significance of our findings by showing increased miR34a and decreased Ang1 expression in 3 independent cohorts of human lung samples.

BPD has been getting more attention as neonatologists from across the world are getting better in saving the lives of premature neonates. This has mobilized intense research to fill the knowledge gaps in all aspects of this dreadful disease. The hallmarks of BPD are lung inflammation and cell death leading to impaired alveolar development and dysregulated vasculogenesis. Laboratories focused on BPD research have been attempting to target the major pathophysiological processes to cure or treat BPD, but have had limited success, to date. miRs are small molecules (~22nt) that target multiple mRNAs, simultaneously. Multiple miRs have been implicated in several diseases, the predominant one being cancer. Although we had come across several papers that showed the importance of miRs in many respiratory diseases (chronic obstructive pulmonary disease or COPD, asthma, emphysema), we were not aware of miR34a being associated with BPD until we saw a robust and consistent increase of this molecule in the miR microarray screen in our HALI and BPD mice models. Other investigators have also noted increased miR34a expression in rat and mouse models of HALI and in human asthma (to which BPD patients may be predisposed to in the long-term).

BPD is a multifactorial disease with genetic-environmental interactions contributing to its pathogenesis. It was first described by Northway *et al* in 1967 in relatively late-gestation preterm neonates who were invasively ventilated and exposed to high concentrations of supplemental oxygen (hyperoxia) for prolonged periods to treat respiratory distress syndrome (RDS). These infants had airway injury and parenchymal fibrosis caused by oxygen and invasive mechanical ventilation. The transition from *in utero* life from a hypoxic environment into room air (RA) at birth, is a relatively hyperoxic event in the life of a newborn, even for a term infant with mature lungs. With the advancement of technology and improvement in medical management, the pathology of BPD has changed over the years. BPD is now a disease of infants born even more prematurely at <= 28 weeks of gestation with birth weights of <= 1000g. The pulmonary phenotype of BPD is characterized by large, simplified alveoli, disorganized blood vessels, with minimal/variable fibrosis. Inflammation remains a key contributor to the pathogenesis of BPD. Newborn mouse models of BPD have been created by exposing neonatal pups to 40, 60, 80-100% O_2_ after birth for the first 4 postnatal (PN) days (saccular stage of lung development), followed by a recovery period of 10 days (alveolar stage of lung development) in RA to mimic mild, moderate and severe BPD in human preterm infants. The combination of a period of hyperoxia-induced injury and repair, concomitant with on-going lung development, is what distinguishes the final pathology of BPD from other pulmonary disorders.

In our recent study, we reported for the first time that hyperoxia-induced stress is *‘the’* contributing factor in BPD that impairs the balance of miR34a and its target genes. While miR34a was significantly increased in the lungs of mice exposed to hyperoxia in comparison to the RA controls, it was decreased in mice exposed to hypoxia. Furthermore, while there was no change in the expression of miR34a in the lungs of newborns postnatally when pregnant rats were treated with antenatal lipopolysaccharide (LPS) administration (mimicking chorioamnionitis), additional postnatal hyperoxia exposure led to a significant upregulation of miR34a. These results strongly suggest that higher concentrations of supplemental oxygen invoke miR34a in a specific manner in the developing lung. Similarly, while epithelial-mesenchymal transition (EMT) is a characteristic feature of all progressing cancers encompassing a series of phenotypic, biochemical and physiological changes enabling cell spreading, we show that miR34a-driven EMT occurs in the HALI/BPD mice models, as well. Additional forms of lung injury (for e.g. stretch, which occurs during invasive ventilation) that contribute to the pulmonary phenotype of BPD needs to be evaluated in future studies to assess the role of miR34a in these injuries.

As BPD is a complex disease, it involves several cell signaling molecules and pathways. In our publication, we focused primarily on the Angiopoietin1/Tie2 signaling pathway. However, additional downstream targets of miR34a with possible links to BPD, deserve additional study. Sirtuin1 (Sirt1) is a downstream anti-inflammatory protein target of miR34a; we have previously noted that decreased levels of Sirt1 is associated with an increased likelihood of developing BPD in human preterm neonates. Overexpression of Sirt1 provides a cell survival advantage by inhibiting apoptosis, extending the cellular lifespan and resisting senescence. Cellular senescence is a status of irreversible growth arrest which has been considered a hallmark of BPD. Alveolar hypoplasia due to hyperoxia exposure in neonatal mice is also due, in part, to cell cycle arrest secondary to reduced histone deacetylase (HDAC) activity and up-regulation of the senescence markers p16 and p21. Autophagy has recently been implicated in the regulation of hyperoxia-induced epithelial cell death as is the case in BPD. Using pharmacological inhibitors and gene silencing techniques, we have previously reported that the activation of autophagy, upon hyperoxia exposure, demonstrated a protective role with an anti-apoptotic response. Specifically, inhibiting the regulatory associated protein of mTOR (Raptor or RPTOR) in hyperoxia settings as evidenced by wild type (WT) mice treated with Torin2 (a chemical inhibitor of mTORC1, which includes Raptor and mTOR proteins) or administered *Rptor *siRNA via intranasal delivery or *Rptor *(+/-) mice, limited lung injury by enhancing autophagy to sufficient levels, decreasing apoptosis, improving lung architecture and increasing survival. Furthermore, we identified increased protein expression of phospho-Beclin1, LC3-II and LAMP1 suggesting altered autophagic flux in the lungs of human neonates with established BPD.

Thus, miR34a appears to be a "master regulator" which targets multiple pathways already implicated and/or possibly associated with BPD (**Fig. 1**). Identification of a master molecule like miR34a opens the therapeutic possibility to manage as dreadful and devastating a disease as BPD, which could potentially dramatically impact the life of premature babies all around the globe. One of the challenges that drug developers often face with is picking the right target. Most of the time, the limiting factors for the intracellular uptake of a naked gene are its hydrophilicity, negative charge, high molecular weight, elevated hydrodynamic size and the rapid degradation it undergoes caused by serum endonucleases and the reticuloendothelial system. Hence, the ideal approach is based on the use of polymeric nanoparticles, which have been demonstrated to be suitable for a wide range of applications for the delivery of potential drugs. These nanoparticles have the aim of improving the biopharmaceutical features of the encapsulated drug as well as its sustained release. However, these approaches have not yet been proven effective for pulmonary diseases. In our BPD modeling system, we administered the miR34a inhibitor intra-nasally to the neonatal pups at a dose of 20 μM for 2 days (at PN2 and PN4), which was well tolerated by the pups without any visible side effects. The reason we preferred the intra-nasal method was because, as BPD is a disease of the lungs, we wanted to target this organ directly, with minimal loss of our therapeutic compound. We believe that pulmonary delivery of a miR34a inhibitor could be enhanced by combining it with a delivery vehicle such as exogenous surfactant. Exogenous surfactant delivery (either via an endotracheal tube or using less-invasive methods) is a standard-of-care approach for the management of infants with RDS, and the timing of delivery of surfactant coincides with our experimental data that early and short-term inhibition of miR34a will result in a dampening of damaging cell signaling pathways and enhancement of lung development ("healing") towards normalcy, rather than "repair" (BPD). Furthermore, since the Type II pneumocytes actively re-cycle surfactant (endogenous and exogenous), the drug would enter the target cell. Hence, this approach will allow the drug to reach the alveolar compartment swiftly and in adequate quantities, minimizing off-target effects including potential toxicities.

**Figure 1:**
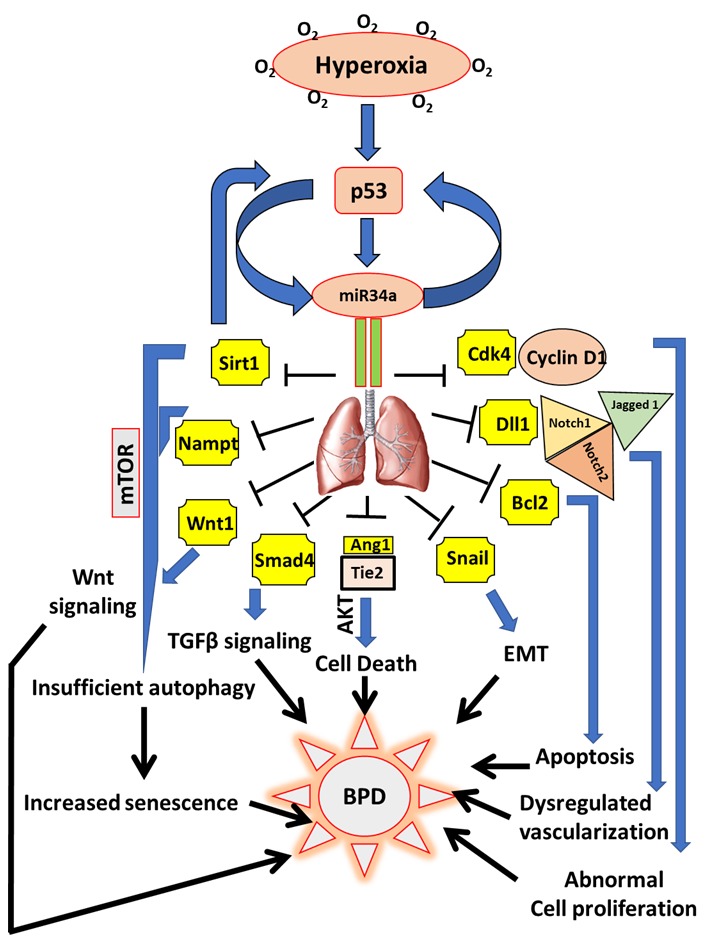
FIGURE 1: miR34a as a master regulator of the proposed cell signaling pathways involved in the pathogenesis of BPD. Hyperoxia exposure to the developing lungs stimulates p53 to activate miR34a in a positive feedback loop. In the lungs, miR34a targets a host of genes which exert their effects either singly or in combination with other genes and signaling pathways that eventually causes BPD in neonates. Upon being targeted by miR34a, Sirt1 is decreased, which leads to an increase in mTOR (a negative regulator of autophagy), that results in insufficient autophagy causing enhanced senescence and impaired alveolarization. Decrease in Sirt1 also increases p53 in a negative feedback loop. Nampt is another target of miR34a which may act in association with Sirt1 to induce senescence. Decrease in Wnt1 and Smad4 interferes with the Wnt and TGFβ signaling pathways responsible for overall lung development/response to hyperoxia. By downregulating Ang1 and its receptor, Tie2, miR34 induces cell death through the Akt pathway. Epithelial-mesenchymal transition (EMT) is brought about by the inhibition of Snail 1, apoptosis by Bcl_2_, impaired vascularization through inhibition of Dll1 and its receptors Notch1, Notch2 and Jagged 1, and abnormal cell proliferation by inhibition of the Cyclin1-Cdk4 complex. All the above pathophysiological processes that are targeted by miR34a are involved in the pathogenesis of BPD. Ang1: angiopoetin1; BPD: bronchopulmonary dysplasia; miR: microRNA; mTOR: mechanistic target of Rapamycin; Nampt: Nicotinamide phosphoribosyltransferase; Sirt1: Sirtuin1; TGFβ: transforming growth factor beta.

We hope that the knowledge gained regarding the role of miR34a in the pathogenesis of BPD from our publication will spur scientific progress in understanding the pathogenesis of BPD, as well as inspire investigators to provide therapeutic insights.

